# Investigating the Relationship Between Sleep Quality and Sociodemographic, Lifestyle, and Psychological Factors in Bosnian Young Adults

**DOI:** 10.1192/j.eurpsy.2025.2337

**Published:** 2025-08-26

**Authors:** F. Obuća, O. Aydın

**Affiliations:** 1 International University of Sarajevo, Sarajevo, Bosnia and Herzegovina

## Abstract

**Introduction:**

Sleep quality is a critical component of overall well-being, yet it is influenced by a variety of factors, including sociodemographic characteristics, lifestyle habits, and psychological conditions. Despite its importance, research on sleep quality among Bosnian young adults is scarce, making this study particularly valuable in filling that gap.

**Objectives:**

This study aimed to investigate the relationship between sleep quality, as measured by the Pittsburgh Sleep Quality Index (PSQI), and a range of sociodemographic, lifestyle, and psychological factors in a sample of Bosnian young adults.

**Methods:**

A total of 283 Bosnian young adults were enrolled in the study through convenience sampling. The study assessed sociodemographic factors (age, gender, education level, religion, employment, residential area, marital status, income level), lifestyle factors (use of electronic devices before bedtime, daytime napping habits, exercise level, smoking, alcohol consumption, drug abuse, caffeine intake, diet, exposure to natural light, consistent sleep schedule), and psychological factors (mindfulness measured by the Five Facet Mindfulness Questionnaire (FFMQ), and stress, depression, and anxiety measured by the Depression Anxiety Stress Scales (DASS-21)). Descriptive statistics, correlation analysis, and multiple linear regression were used to analyze the data.

**Results:**

The findings indicate that sleep quality is significantly predicted by several factors. Negative predictors of sleep quality included the Observe facet of mindfulness and maintaining a balanced diet. Conversely, positive predictors that were associated with poorer sleep quality included higher levels of stress, smoking, and the use of electronic devices before bedtime.

**Conclusions:**

The study highlights the complex interplay between sociodemographic, lifestyle, and psychological factors in determining sleep quality among Bosnian young adults. These findings underscore the need for targeted interventions that address these specific factors to improve sleep quality in this population.

**Disclosure of Interest:**

None Declared

